# Carded Tow Real-Time Color Assessment: A Spectral Camera-Based System

**DOI:** 10.3390/s16091404

**Published:** 2016-08-31

**Authors:** Rocco Furferi, Lapo Governi, Yary Volpe, Monica Carfagni

**Affiliations:** Department of Industrial Engineering, University of Florence (Italy), Firenze 50139, Italy; lapo.governi@unifi.it (L.G.); yary.volpe@unifi.it (Y.V.); monica.carfagni@unifi.it (M.C.)

**Keywords:** spectrophotometry, spectral camera sensor, carding process, color assessment

## Abstract

One of the most important parameters to be controlled during the production of textile yarns obtained by mixing pre-colored fibers, is the color correspondence between the manufactured yarn and a given reference, usually provided by a designer or a customer. Obtaining yarns from raw pre-colored fibers is a complex manufacturing process entailing a number of steps such as laboratory sampling, color recipe corrections, blowing, carding and spinning. Carding process is the one devoted to transform a “fuzzy mass” of tufted fibers into a regular mass of untwisted fibers, named “tow”. During this process, unfortunately, the correspondence between the color of the tow and the target one cannot be assured, thus leading to yarns whose color differs from the one used for reference. To solve this issue, the main aim of this work is to provide a system able to perform a spectral camera-based real-time measurement of a carded tow, to assess its color correspondence with a reference carded fabric and, at the same time, to monitor the overall quality of the tow during the carding process. Tested against a number of differently colored carded fabrics, the proposed system proved its effectiveness in reliably assessing color correspondence in real-time.

## 1. Introduction and Background

One of the most relevant parameters to be taken into account when designing yarns is color, especially when dealing with wool fabrics obtained by weaving fancy yarns. Fashion designers, in fact, usually conceive fabrics whose color evokes most recent trends in fashion market; consequently, textile companies are required to accurately reproduce such desired colors. Usually, the final product is obtained by mixing together several pre-colored fibers to create a mixture, named “blend”, whose color resemble the desired one [1]. Therefore, textile company colorists are required to choose the correct amount of differently pre-colored fibers to be mixed together for obtaining a desired final blend.

**Figure 1:**
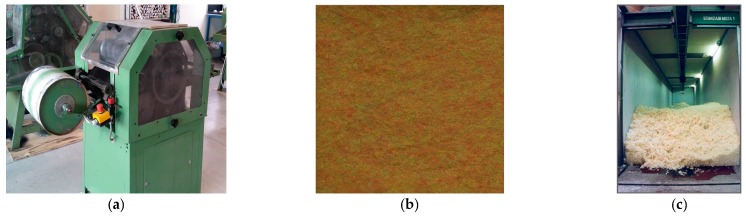
(**a**) Small drum carding machine used to create carded fabric samples to be inspected by colorists; (**b**) example of a carded fabric obtained by using the small drum carder; and (**c**) blow room where fibers are opened, cleared and mixed.

Their work, mainly based on experience, is aided to a certain extent by the availability of databases where spectrophotometric information regarding pre-colored fibers is stored together with sets of suggested percentages to be used for obtaining given color targets (i.e., with the so called “recipes”). Depending on color desired by e.g., the fashion designer, usually materially exemplified by a sample of colored fabric named “reference fabric”, textile colorists start to search their storehouse for correctly pre-colored fibers. Afterwards, they mix them (in laboratory) according to the given recipe, by using a small drum carding machine (like the one shown in Figure 1a) providing, as a result, a carded fabric sample (see Figure 1b).

The sample is then compared with the reference one, with the aim of determining the color difference between them. The comparison is performed by using spectrophotometers working under several color spaces, such as CIE 1976 color space (CIELAB), or using color tolerance systems such as the quasimetric Colour Measurement Committee defined ones (CMC (2:1)) [2] and using a number of standard illuminant such as D65 or TL84 [3]. In case the colorimetric distance between laboratory sample (carded fabric) and reference ones exceeds a given threshold (e.g., DE_CMC(2:1)_ > 0.8), colorists are required to slightly change the recipe to match the desired color. This usually entails the need of producing several samples in order to progressively reduce the gap between the color of the final product and the desired one. Once the color of the obtained blend is considered to be sufficiently close to the target one (often after a number of trials), the percentages of pre-colored fibers used to create the blend are stored and used for manufacturing the yarn at industrial scale.

**Figure 2:**
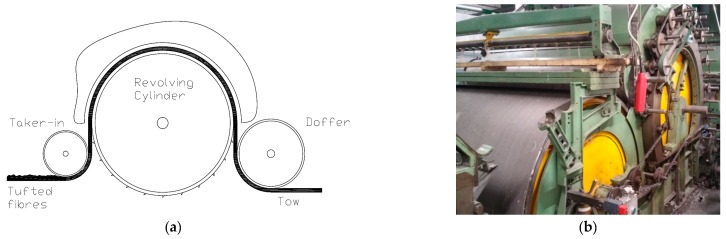
Carding process: (**a**) Scheme of working station (module) consisting on taker-in, revolving and doffer cylinder; and (**b**) actual working station.

To this purpose, a large amount of pre-colored fibers mixed according to the modified recipe obtained according to the process described above, are sent to a blow room (see Figure 1c) where fibers are opened, cleaned and mixed together into a fibers agglomerate called “tuft”. Subsequently, the tuft feeds a carding machine. As widely recognized [4], carding is one of the most important operations in the entire yarn cycle production process as it impacts the final features of the yarn [5]. In detail, carding allows opening the tufts into individual fibers, disentangling them and eliminating all the impurities contained in the fiber including possible small fibers agglomerates (called “neps”). Moreover, it allows to stretch the fibers and to make them parallel to each other with the final effect of transforming the tufted fibers into a regular mass of untwisted fibers, named “tow” [6]. Carding machines are able to create the tow thanks to a number of modules disposed in series. Each module consists of a tacker-in cylinder followed by a revolving cylinder covered with fine hooks; in its turn, this revolving cylinder is followed by a doffer [7] (see Figure 2).

After the mass of untwisted fibers passes through the taker-in, it is processed by the revolving cylinder and its fibers are therefore arranged in a series of entangled bundles of fibers, approximately parallel to each other. Thus, the fibers form a small thickness planar web.

**Figure 3:**
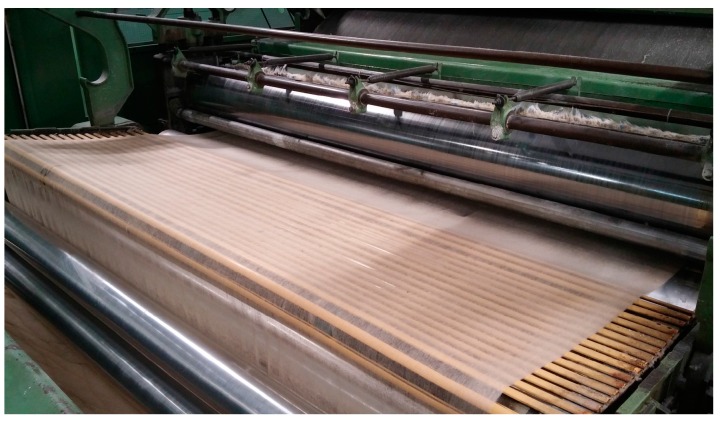
Final tow to be sent to the drawing.

This process is repeated in the remaining modules thus forming the “final” tow (Figure 3).

**Figure 4:**
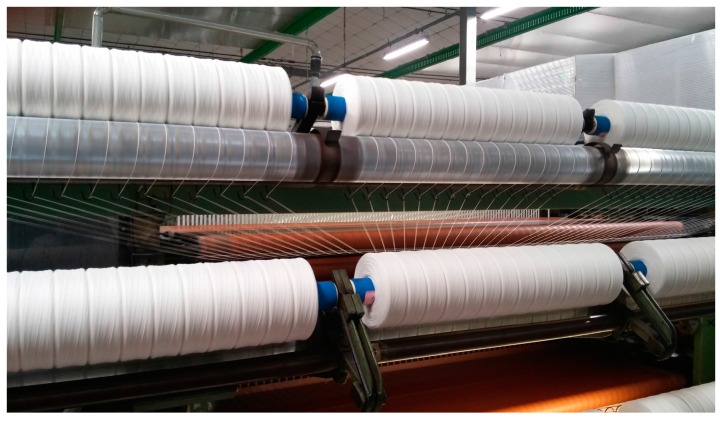
Untwisted yarn obtained by the tow, wrapped in spools.

Afterwards, the tow undergoes a drawing process so that the loose rope of carded fibers are converted into a thinner rope until it becomes, in practice, an untwisted yarn. Such a yarn is, finally, wrapped in a spool (see Figure 4) and eventually sent to the spinning machine to confer the final properties such as strength, length, twist yarn, yarn count and fineness.

The entire process described above (comprising both the laboratory testing phase and the industrial production one) is prone to the following issues when dealing with color reproduction:

(1) Often not even raw materials are colored as expected; as a consequence, at a laboratory level, when blending differently colored fibers (even according to well established recipes) the final resulting blend may be very different from the reference (target). As mentioned above, this requires colorists to iterate the fabric blend creation a number of times before reaching the desired color.
(2) At an industrial scale, when dealing with carding process it is not possible to guarantee the correspondence between the tow color and of the one of the laboratory carded blend. This is mainly due both to scaling proportions of fibers from the laboratory scale to the industrial one and to the blowing machine itself that commonly removes small fibers together with impurities, thus changing the actual percentage of pre-colored fibers.
(3) At an industrial scale, when dealing with the spinning process, the final product (yarn) has completely different shape from the carded fabric one; as a consequence its color can be considerably different from the lab sample one. Moreover, the yarn travels at high speed (more than 200 m/min) thus making a real-time measurement particularly difficult. In addition, it has to be considered that the yarn undergoes a weaving process finalized to the creation of knitwear; therefore, the weaved fabric color itself could result far to be similar to the reference one, measured on a carded fabric.

The solution to issue (1) is still a hot topic today in the field of textile color control, as demonstrated by recent studies [8,9,10,11]. In fact, to speed-up the process of reproducing a target color using laboratory carded blends, several computer-based approaches have been proposed in literature, mainly dealing with the spectrophotometric prediction of dyed fabrics and with particular focus on the study of the color mixing model. These methods can be divided into two categories: theoretical methods and ANN-based ones. Theoretical methods are mostly based on the widely known Kubelka–Munk (K-M) theory [12,13,14,15], generally used for the analysis of diffuse reflectance spectra obtained from weakly absorbing samples. These methods perform well when dealing with fabrics obtained by mixing up to 4–5 differently colored fibers [12]. Referring to ANN-based methods, they provide a reliable and very practical approach for helping the colorist in color matching when the number of pre-colored fibers is higher than 5. This is demonstrated in [8] where a transfer function linking the color spectrum obtained by a linear combination of the spectra of each component with the measured reflectance values of a first-attempt blend is determined. In practice, using techniques such as the ones mentioned above, it is possible to strongly reduce the number of trials to determine the optimal recipe for obtaining a desired color.

Issue (3) has been partially overcome in [16] where a direct yarn color measurement and reproduction method based on a multispectral imaging system is proposed. The system compares the in-line measurement of the yarn color, obtained with a spectral camera, with the laboratory static measurement. The measured color differences ranges from 0.83 to 2.48, obtaining a data dispersion of 0.49 CMC(2:1) units and a data mean of 1.41 CMC(2:1). These results show the effectiveness of spectral cameras for real-time measurement of yarn color (even if no measurement of the correspondence between yarn color and reference color-carded blend has been addressed so far).

Unfortunately, no method for measuring in real time the color of the tow and to compare it with the reference one has been developed yet. As a consequence, issue (2) still remain a major problem for textile companies.

On the basis of the above considerations, and taking into account the actual maturity of sensors for textile industry [17], the main aim of the present work is to provide a spectral-camera-based system (hardware + software) able to perform a real-time measurement of a carded tow to assess the color correspondence with a reference carded fabric and to monitor, at the same time, the overall quality of the tow during the carding process. In particular, such a system is meant to work by examining a continuous tow of fibers whose composition is selected according to a hypothetic company's recipe. The authors will also demonstrate that the devised system allows a reliable color control and thus is capable to provide an effective solution to issue (2).

The remainder of the paper is structured as follows: in Section 2, the laboratory testing system for determining the optimal recipe of a carded fabric and the real-time color measurement system addressed to quality control of the tow are described. In Section 3, the entire color control process is described with reference to a case study. In Section 3, a discussion of experimental results obtained by the proposed system is provided in order to highlight pros and cons of the system itself.

## 2. Materials and Methods

As stated above, the final aim of the present work is to provide a system able to perform a real-time colorimetric assessment during the carding process. Accordingly, the following tasks had to be accomplished to create such a system:

(1) definition of a laboratory setup able to perform repeatable color measurement on both the reference fabric and on the carded blend used for determining the optimal recipe; and
(2) definition of the real-time color measurement system (hardware + software) for assessing quality control of the carded tow.

### 2.1. Laboratory Testing System

The definition of a laboratory testing system is crucial to guarantee a repeatable color measurement on both the reference fabric and on the carded blend used for determining the optimal recipe. In this work, the laboratory equipment consists of: (1) the small drum carder machine in Figure 1a, fed by the raw materials (pre-colored fibers) so as to obtain a homogeneous fabric like the one depicted in Figure 1b; and (2) a Hunterlab Ultrascan VIS reflectance spectrophotometer to be used for measuring the spectral response of both the reference fabric and the carded one.

In detail, once the carded fabric is obtained, the spectrophotometer is used to measure its light reflectance in the wavelength range of 400 nm to 700 nm, with a step equal to 10 nm (that is the actual standard wavelength step adopted by textile companies). The resulting spectrum, consisting of 31 reflectance values, is obtained using a scattered light measurement in SCE mode (specular component excluded). The scan is made with a neutral white background, using an eight-degree angle between the light source (D65 illuminant) and the sample, according to textile standards [8]. A zero calibration is used to compensate for the effects of stray light due to the changing flare characteristics of the optical system. Such a calibration is performed by removing the spectrophotometer protective cap from the aperture and aiming the aperture into the air, so that no objects are within 1 m and no light source is aimed at it. The white calibration of the spectrophotometer, used to set the maximum reflectance to 100%, shall be performed at the beginning of data acquisition using a standard “white” cap whose reflectance is known to be equal to 1.

**Figure 5:**
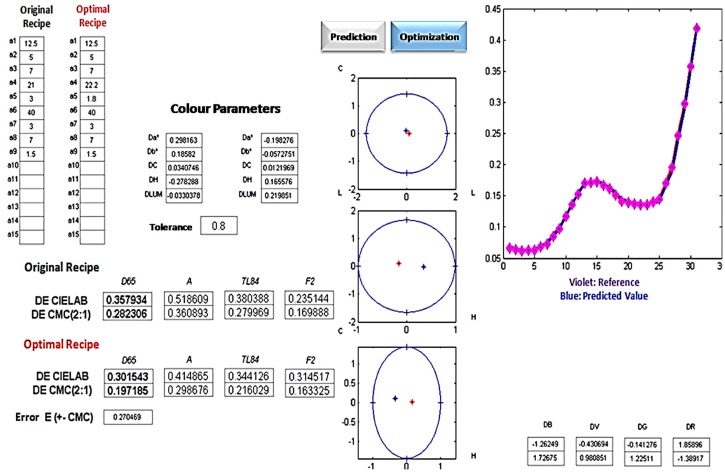
System used for laboratory testing [1] implementing algorithms proposed in [8].

Once acquired under controlled conditions (i.e., environmental temperature in the range 23 ± 1 °C and SCE mode on the spectrophotometer), the actual cap spectral response is used to normalize the fabric acquisitions i.e., to obtain new measured reflectance varying in the range 0–1. Acquired data can be stored in a PC in the form of a 31-elements vector representing the reflectance values vs. the wavelength for an examined sample. To ensure adequate robustness and representativeness of results, the largest (25.4 mm^2^) measuring area of the spectrophotometer shall be selected. Once the reflectance values of carded fabric are available, it is possible to evaluate the colorimetric distance with respect to the desired reference. In case the colorimetric distance is higher than a desired value (usually equal to 0.8) the recipe should be slightly changed. Despite commercial spectrophotometers are usually equipped with a software package able to determine color distances between two samples, in the present work the system developed in [1] is used for this purpose. In fact, besides providing CIELAB and CMC(2:1) distances, such a system (see Figure 5) implements the algorithms devised in [8] which allow the user to determine an optimal recipe for a given carded fabric.

In other words, the method devised in [8] allows determining the optimal recipe $A=\left[\alpha_{1},\alpha_{2},\ldots ,\alpha_{n}\right]$ (where $\alpha_{i}$ is the percentage of the ith pre-colored fiber composing the blend and where $\sum_{i=1}^{n}\;\alpha_{i}=1$), for obtaining a carded fabric whose color is as close as possible to the reference one. Moreover, the laboratory equipment is able to provide, in output, the following parameters:

- the spectral response of carded fabric $R_{c}\left(\lambda \right)$;
- the spectral response of the reference fabric $R_{r}\left(\lambda \right)$; and
- the colorimetric distance ${\text{DE}}_{c-r}$ between the carded and the reference fabric.

### 2.2. Real-Time Color Measurement System for Quality Control of a Carded Tow

As mentioned before, the real-time system comprises a hardware part and a software one, and the following two sections provide a detailed description of them.

#### 2.2.1. Hardware Setup

Due to the structure of the carding machine and, more specifically, to the tow flow during the process, a very practical solution is to acquire color information along transversal sections of the tow, as a consequence the most suitable sensor is a linear spectral camera.

The selected device is a Middleton DV Spectral Camera VNIR consisting of an imaging spectrograph (ImSpector V10E, Specim, Spectral Imaging Ltd, Oulu, Finland) and a monochrome camera equipped with a interline Kodak KAI-1020 CCD sensor (Eastman Kodak Company, New York, NY, USA) with pixel size 7.4 µm × 7.4 µm. This device provides, as an output:

- up to 30 fps each frame consisting on a monochrome image of the scene at a resolution of 1600 ppi and spatial resolution (RMS) lower than 9 µm; and
- the reflectance factors in the spectral range 400–1000 nm (with a minimum spectral resolution equal to 2.8 nm) for 1200 points measured in the inspected line. Declared spectral sampling varies in the range 0.72–5.8 nm/pixel and numerical aperture of the VNIR detector is F/2.8.

**Figure 6:**
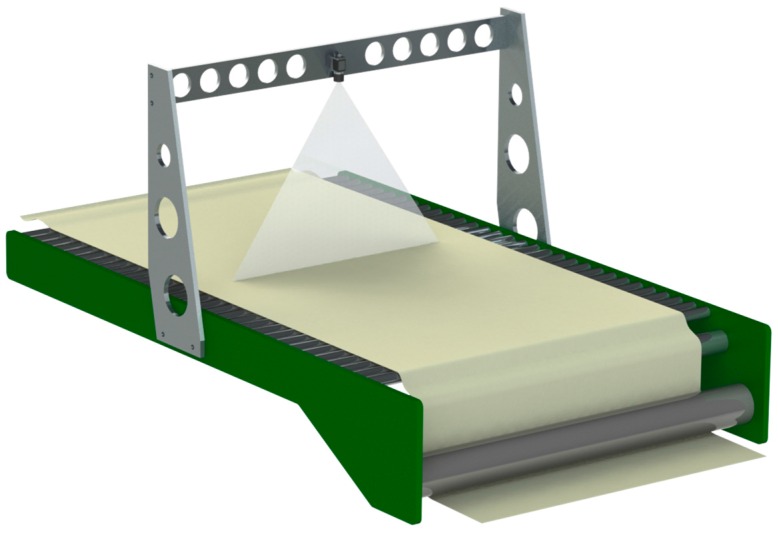
Architecture of the acquisition system. Due to confidentiality agreement, only a scheme of the system can be shown.

The sensor is attached to a metallic frame in order to acquire the tow at a distance of approximately 500 mm. The frame is positioned downstream of the last section (taker-in–revolving cylinder–doffer) and immediately upstream of the drawing machine (see Figure 6).

With this architecture, the acquisition system is able to acquire a tow line of 600 mm length for each frame. A zero calibration is used to compensate for the effects of stray light due to the changing flare characteristics of the optical system.

White and black calibrations of the camera are performed at the beginning of data acquisition. First, the spectral response w(λ) of a standard ceramic cap is measured using the spectral camera. Then, the camera lens is clogged by using a Schott RG-1000 filter, thus acquiring a spectral response d(λ).

Finally, the actual spectral response $R_{T}\left(\lambda \right)$ for a given acquisition can be derived by the following equation:

(1) $$R_{T}\left(\lambda \right)=\frac{R_{sample}\left(\lambda \right)-d\left(\lambda \right)}{w\left(\lambda \right)-d\left(\lambda \right)}$$

where $R_{sample}\left(\lambda \right)$ is the reflectance value of the sample directly measured by the spectral camera.

Considering an average speed of the tow during the carding process of about 0.10 m/s, by setting the frame rate equal to 25 fps it is possible to acquire a single tow section (i.e., a line of 1600 pixels) every 0.4 cm. At the same time, for each section of the tow, the spectral reflectance of 1200 equidistant points (in the range 400–1000 nm) can be acquired. This means that the device has the capability of acquiring the spectral reflectance of a point every 0.5 mm. Finally, by setting the spectral resolution equal to 10 nm, measured spectra will consists of 61 reflectance values.

Another important aspect to be taken into account for conceiving the acquisition system is the choice of a proper illuminant when acquiring the tow with the spectral camera. In the proposed system, a CIE Standard Illuminant D65 lamp (with a temperature of 6504 K, roughly corresponding to the average midday light in Western Europe/Northern Europe), commonly used in the textile field for color assessment, is used. This illuminant has the double advantage of illuminating the inspected surface with a rich diffusive light and of allowing the use of standard colorimetric equations [18] for evaluating color.

Finally, acquired data are transferred to a PC using a standard Giga-Ethernet interface.

#### 2.2.2. Software Parameters

Data acquired with the previously described sensor (e.g., the 1200 reflectance values measured along a tow section of 600 mm every 1/25 s) provide relevant information regarding tow actual color, as described in the following. Let ${R_{T}}^{j}\left(t\right)$ be the reflectance value of the j-th of the 1200 points acquired at an instant t after the carding process is started. The average spectral response of the analyzed section can be easily evaluated as follows:

(2) $${\text{avgR}}_{T}\left(t,\lambda \right)=\sum_{j=1}^{1200}\frac{{R_{T}}^{j}\left(t,\lambda \right)}{1200},\;\text{with}\;\lambda \in \left[400\;\text{nm};700\;\text{nm}\right]$$

Each reflectance value derived by Equation (2) provides the “actual spectral response” of the tow in a given section. As better explained below, this can be compared, limitedly to the reflectance values in the visible range 400–700 nm, either with the spectral responses of carded fabric $R_{c}\left(\lambda \right)$ or with the spectral response of the reference fabric $R_{r}\left(\lambda \right)$ depending on the company preference. Moreover, by defining a time interval Δt and a step parameter k (with k∈ℕ), it is possible to compare the actual measurement of the tow at every instant t+kΔt, i.e., ${\text{avgR}}_{T}\left(t+k\Delta t,\lambda \right)$, with ${\text{avgR}}_{T}\left(t,\lambda \right)$ thus allowing a quality control over the entire tow during carding process.

In order to compare the above-mentioned reflectance values, it is first necessary to introduce the color distance commonly used by textile companies, which is CMC(2:1) [18,19]. Such a distance is given by the following equation:

(3) $${\text{DE}}_{\text{CMC}\left(2:1\right)}=\sqrt{{\left(\frac{\Delta L}{{\text{lS}}_{L}}\right)}^{2}+{\left(\frac{\Delta C}{{\text{cS}}_{c}}\right)}^{2}+{\left(\frac{\Delta H}{S_{H}}\right)}^{2}}$$

where:

- $\Delta L=L_{\text{reference}}-L_{\text{sample}}$ is the difference, in terms of luminance (L) value, between the reference and the sample;
- $\Delta C=C_{\text{reference}}-C_{\text{sample}}$ is the difference, in terms of chroma (C) value, between the reference and the sample;
- $\Delta H=H_{\text{reference}}-H_{\text{sample}}$ is the difference, in terms of hue angle (H), between the reference and the sample;
- $S_{L}$, $S_{c}$ and $S_{H}$ are the semi-axis of the CMC ellipsoid whose centre is defined by the triplet of LCH values of the reference sample; and
- l and c are empirical correction factors; for CMC(2:1) distance l=2 and c=1.

**Figure 7:**
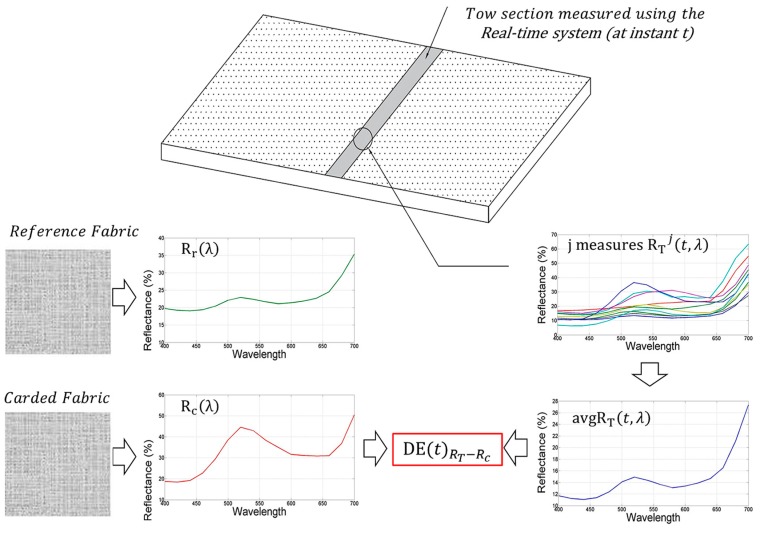
Measurement of the CMC(2:1) color distance between Carded fabric, measured in Lab, and tow measured at instant *t* using the real-time colorimetric system.

**Figure 8:**
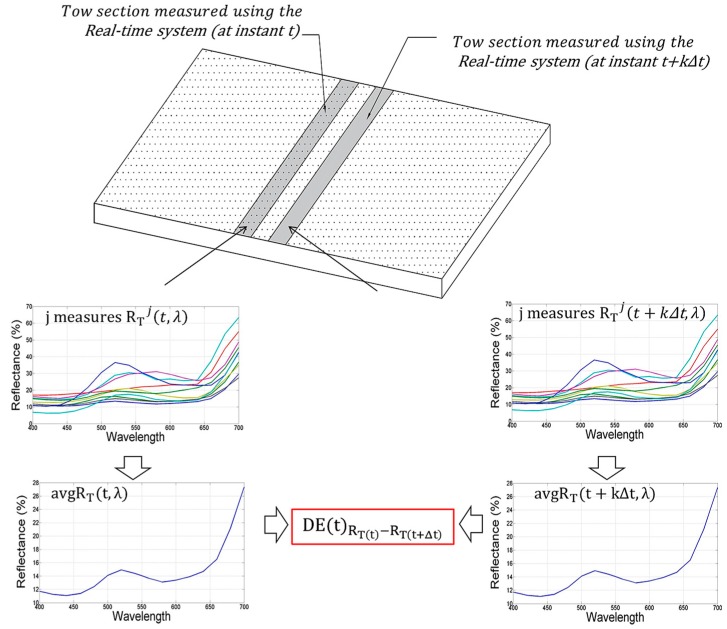
Measurement of the CMC (2:1) color distance between two measurements of the same tow assessed in different instants (t and t+kΔt).

**Figure 9:**
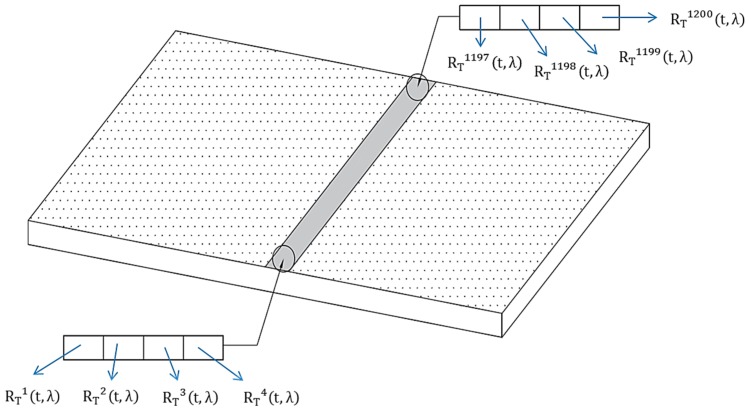
Different reflectance measurements on different points on a single tow section.

For this purpose, the acquired parameters ${\text{avgR}}_{T}\left(t,\lambda \right)$, ${\text{avgR}}_{T}\left(t+\Delta t,\lambda \right)$ and $R_{c}\left(\lambda \right)$ (or $R_{r}\left(\lambda \right)$ if preferred by the company colorists) are first converted in the XYZ tristimulus [20]; then, the XYZ values are converted in the CIELCH space simply using the XYZ to CIELCH relations [21] taking into account the standard D65 illuminant properties. Finally, using Equation (3), it is possible to define the following parameters:

1. CMC(2:1) Colorimetric distances $\text{DE}{\left(t\right)}_{R_{T}-R_{c}}$ between ${\text{avgR}}_{T}\left(t,\lambda \right)$ and $R_{c}\left(\lambda \right)$; these distances are defined at the generic instant t during the carding process and allow to monitor how, averagely, the color of the tow changes with respect to the reference fabric (see Figure 7). Of course, in the case that the comparison between ${\text{avgR}}_{T}\left(t,\lambda \right)$ and $R_{r}\left(\lambda \right)$ is preferred to the comparison between ${\text{avgR}}_{T}\left(t,\lambda \right)$ and $R_{c}\left(\lambda \right)$, it is to be taken into account that the color distance $\text{DE}{\left(t\right)}_{R_{T}-R_{r}}$ between them is provided by:
  (4) $${\text{DE}}_{R_{T}-R_{r}}\left(t\right)=\text{DE}{\left(t\right)}_{R_{T}-R_{c}}+{\text{DE}}_{c-r}$$
2. CMC(2:1) Colorimetric distances $\text{DE}{\left(t\right)}_{R_{T\left(t\right)}-R_{T\left(t+\Delta t\right)}}$ between ${\text{avgR}}_{T}\left(t,\lambda \right)$ and ${\text{avgR}}_{T}\left(t+k\Delta t,\lambda \right)$. Once the averaged spectral response of the tow in an instant t is measured, it is possible to monitor how color changes during the carding process (see Figure 8). As a consequence, this parameter measures the local variability of the tow color during the carding process. In fact, this distance is defined at the generic instant t+kΔt.
3. Colorimetric distance between different points on a single tow section. ${R_{T}}^{j}\left(t,\lambda \right)$ values can be directly used to assess differences, in terms of spectral response, in different points of the tow section under inspection (see Figure 9). In this last case, the comparison could be properly performed by comparing spectral response directly, since a common practice for textile field does not exist for this specific case; thus no need of color space conversion is necessary. However, also in this case, the definition of the CMC color distance allows reducing the comparison of 31 reflectance values for each acquired spectrum to a single parameter, thus simplifying the comparison task.

#### 2.2.3. Software Implementation

With the aim of helping the colorists in performing color quality control, a software package was developed using C++ language. Such a package, embedded into a complex ERP software (PROTEX ERP [22], Computer House, Prato, Italy), consists of:

- A library for managing spectral scanner on/off and parameters, D65 lamp on/off and calibration.
- A module for the evaluation of color distances according to Equations (1)–(4).
- A GUI for setting a number of parameters, such as threshold values for CMC distance acceptability, company id code and description of the lot under inspection.
- A module for storing reflectance data and color distances in a SQL database to be accessed by any PC connected to the company network.

Data are processed (acquisition task + color classification) in about 1.2 s using a standard PC equipped with an Intel^®^ Core™ i7 (Intel Corporation, Santa Clara, CA, USA) and 32 Gb RAM (Micron Technology, Boise, Idaho, USA).

## 3. Results

**Figure 10:**
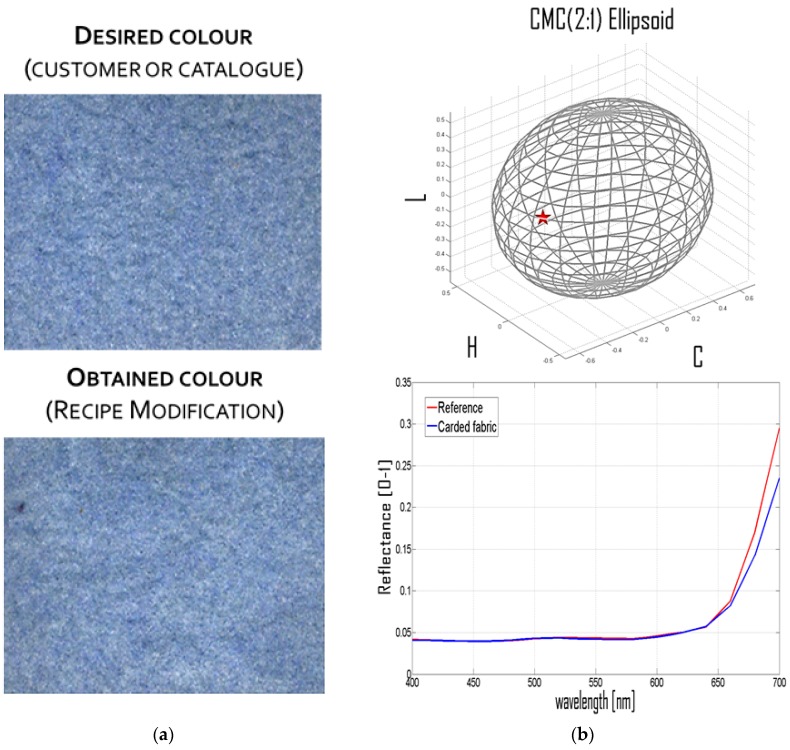
(**a**) Visual comparison between the color of desired and manufactured fabrics; and (**b**) CMC(2:1) Ellipsoid, spectrum $R_{c}\left(\lambda \right)$ and spectrum $R_{r}\left(\lambda \right)$.

To better understand the system workflow, let us consider the entire process to obtain fabric of desired color (see Figure 10).

The fabric is manufactured by an Italian Company named New Mill S.p.a. located in Prato, Italy. By using one of the company’s historical recipes, a first-attempt fabric is obtained using five pre-colored fibers according to the percentages listed in the central part of Table 1.

### 3.1. Laboratory Testing System Results

Using the equipment and method described in Section 2, it is possible to measure the color difference in terms of CIELAB between the reference and the first attempt carded fabrics so as to perform recipe correction. The final result consists of a modified recipe. The use of the new recipe allows obtaining a fabric whose color is very similar to the desired ones (see Figure 10a). Such a modified recipe is shown in the right part of Table 1. In Figure 10b, the CMC(2:1) ellipsoid and the two spectra $R_{c}\left(\lambda \right)$ and $R_{r}\left(\lambda \right)$ are shown, demonstrating the effectiveness of the recipe correction.

### 3.2. Real-Time Color Measurement

**Figure 11:**
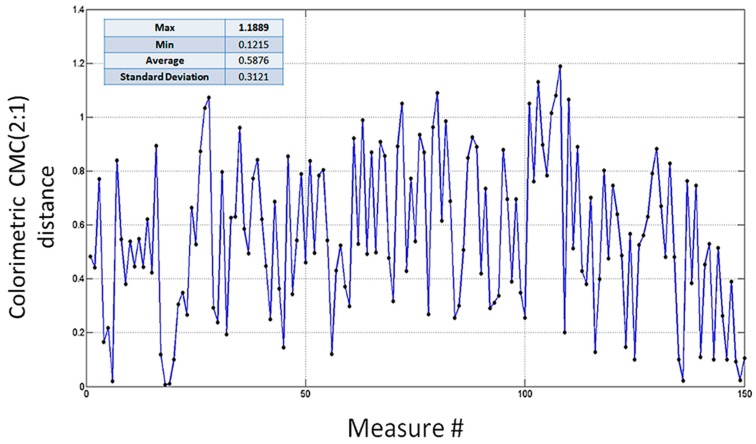
Distances $\text{DE}{\left(t\right)}_{R_{T}-R_{c}}$ measured during the carding process.

**Figure 12:**
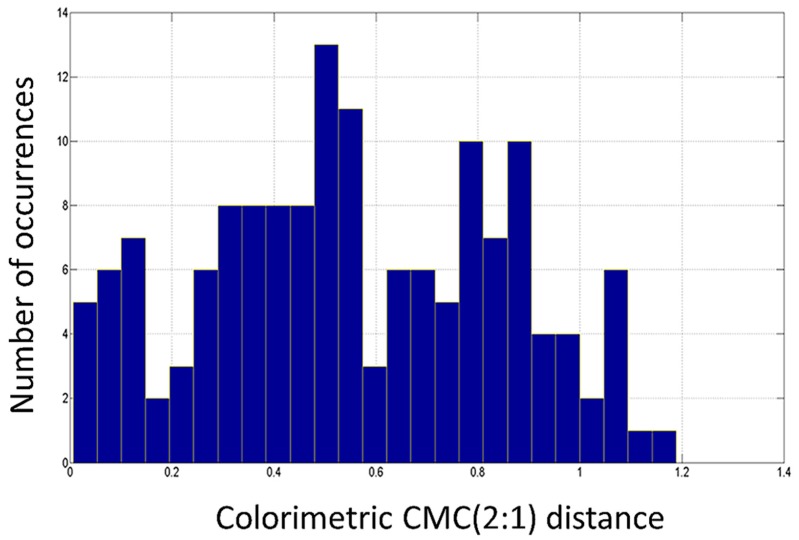
Histogram representing the number of occurrences providing a certain value for the CMC(2:1) distance.

**Figure 13:**
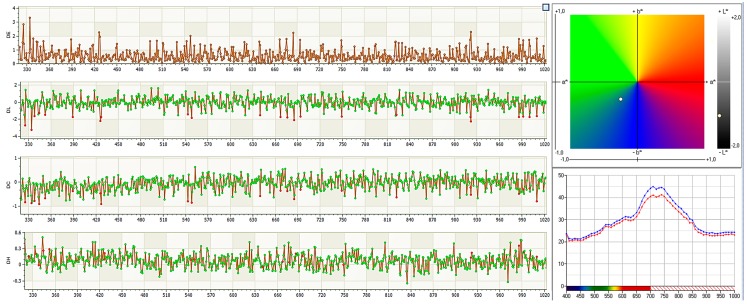
CMC(2:1) distances between the averaged value of spectral reflectance taken each kΔt (with k=1…149).

**Figure 14:**
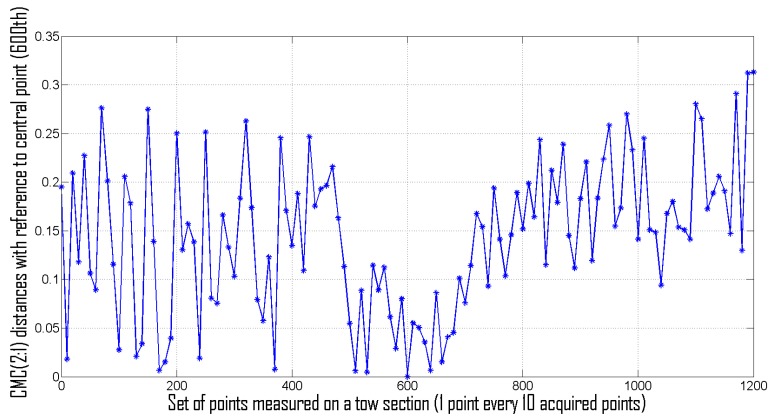
Color distance, in terms of CMC(2:1) between all points of the tow sections and the reference one (central point with index 600).

As stated in Section 2, the machine vision system developed for measuring color of the tow is capable of acquiring up to one image every 0.4 cm. To reduce the amount of data to be processed in real-time, measurement was performed with a time step Δt=2 s so as to measure a single section of the tow every 0.20 m. The entire tow was processed in 300 s; thus, a set of 150 measurements is stored. Using the described system it is possible to measure the following parameters as mentioned in Section 3:

1) Colorimetric distances $\text{DE}{\left(t\right)}_{R_{T}-R_{c}}$ between ${\text{avgR}}_{T}\left(t,\lambda \right)$ and $R_{c}\left(\lambda \right)$. In Figure 11 distances $\text{DE}{\left(t\right)}_{R_{T}-R_{c}}$ measured during the carding process are depicted together with maximum, minimum, average and standard deviation values. In Figure 12 the histogram representing the number of occurrences providing a certain value for the CMC(2:1) distance is shown. By examining Figure 12, it is possible to state that the particular carded tow used of the test has a color distance lower than 0.8 in the 75.33% and lower than 0.9 in 88% of measured sections. Of course it is up to the Company to decide the overall quality of the tow on the basis of these experimental evidences. Referring to the company experimenting the proposed system, the tow is deemed to be of “good quality” since at least 70% of sections presents color distance lower than 0.8 (high quality is CMC(2:1) < 0.8 for 90% of sections). Of course, different companies could decide different quality grades for their own products.
2) Colorimetric distance $\text{DE}{\left(t\right)}_{R_{T\left(t\right)}-R_{T\left(t+\Delta t\right)}}$ between ${\text{avgR}}_{T}\left(t,\lambda \right)$ and ${\text{avgR}}_{T}\left(t+k\Delta t,\lambda \right)$. In Figure 13, take from the proposed system GUI, the CMC(2:1) distances between the averaged value of spectral reflectance taken each kΔt (with k=1…149) are depicted. Figure 13 also shows other related parameters such as, for instance, DC and CH distances [21] and the final measured spectrum. This second modality of color control is considered most relevant for the company staff that tested the proposed system. Their primary interest, in fact, is to guarantee that the entire tow maintains as close as possible the same color during carding process. In this illustrative case, since 97.5% of measures are within a given tolerance (lower than 1.2 in this case), the inspected fabric is considered “high quality” one. In addition, in this case, different companies could decide alternative quality grades for their own products.
3) Colorimetric distance between different points on a single tow section. Let us consider, as an example, the tow section acquired at t=90s; as mentioned above, a set of 1200 ${R_{T}}^{j}\left(90,\lambda \right)$ values can be measured at that time. All these vectors, defining the spectral response of a single equal-spaced pixel of the tow section, can be compared each other in terms of CMC(2:1) distance. Let, now, be ${R_{T}}^{600}\left(90,\lambda \right)$ (i.e., the reflectance of the 600th point—the middle one—of the acquired set) used as reference for monitoring color changes within the tow section. Accordingly, all other spectral responses can be compared with such a point to highlight possible spectral response changes. In Figure 14, the color distance, in terms of CMC(2:1) between a point every 100 acquired points of the tow sections and the reference one, is depicted. Results shows that the color is approximately constant for all central points of the tow section, while distance averagely increases in the margins.

## 4. Discussion

The developed system is currently running at the New Mill S.p.a. Company in Prato (Italy) and was successfully tested with more than 300 carded tows. Assessing the performance of the color control system is not a trivial task since the method described in Section 3 only assesses the quality of carded fabrics using the proposed system and thus does not provide information about the actual performance of the system itself. In order to properly validate the system, a number of tows have been selected as verification samples and a set of additional measurements have been carried out by using workbench spectrophotometer equipping the company laboratory.

More in detail, for a set of 12 tows, the continuous carding process has been stopped every 30 s and data are acquired using the proposed system (10 measurements in total for each of the 12 tows). Then, a portion of the tow (10 mm high) has been cut off and sent to the laboratory to be measured using the spectrophotometer. The spectrophotometric measured reflectance factors $R_{\text{test}}\left(\lambda \right)$ have been then compared, in terms of CMC(2:1) distance, with the parameter ${{\text{avgR}}^{\prime }}_{T}\left(t,\lambda \right)$ obtained by averaging only the points in correspondence of the midline (approx. 5 mm from each border) of the test portion, limitedly to the reflectance values in the visible range 400–700 nm. The results of this comparison are listed in Table 2.

Overall results show that the colorimetric distance between the reflectance acquired with the real-time system and the corresponding reflectance acquired using the spectrophotometer remains lower than 0.35 with an average value equal to 0.3002. These results can be considered satisfactory for the company testing the system and are, in general, comparable to similar systems performing real-time color control in different fields [23].

In any case, from the results listed above, it could be objected that a color difference between static and real-time acquisitions equal to, for instance, 0.3 could make the difference when classifying the quality of the tow. In other words, the same tow could be classified as “high quality” of “average quality” using two different methods. Moreover, while the spectrophotometric method is effectively standardized for textile industry, the use of a real-time quality control system is not a recognized standard. Finally, it has to be considered that the spectrophotometric method implements an integrating sphere consisting of a hollow spherical cavity with its interior covered with a diffuse white reflective coating. This allows acquiring diffusive light. This technique is not easy to be implemented in real-time measurement and positively affects the performance of color measurement.

Accordingly, the main suggestion for companies willing to use the proposed system (or similar approaches based on spectral cameras), is to consider the measured colorimetric distances in relative terms rather than in absolute ones, i.e., to use the proposed method for real time monitoring the process, keeping in mind that the results may differ from the laboratory ones. This is the reason why the company which tested the proposed system opted to use the colorimetric distance $\text{DE}{\left(t\right)}_{R_{T\left(t\right)}-R_{T\left(t+\Delta t\right)}}$ as the main parameter for classifying the tow quality with the proposed system, while maintaining the laboratory spectrophotometric tests in order to assess absolute color correspondence to target.

In any case, to the best of authors’ knowledge, no real-time color control system for carded tows has been presented in scientific and technical literature yet. Therefore, even if the performed color control cannot be as reliable as the spectrophotometric one (at least until a standardization is achieved), obtained results are really encouraging.

## 5. Conclusions

The present work described a spectral-camera based system for investigating the quality, in terms of color, of a tow during the carding process. The system comprises both hardware (sensor, illuminant, and camera support architecture) and software used to process acquired data and to compare them with laboratory-based colorimetric parameters. The system proved to be effective in performing reliable color assessment, especially when the interest is to locally control the color trend during the carding process.

In particular, the system allows:

- comparing the color of the tow during the carding process with respect to the desired fabric;
- locally comparing the overall trend of the tow during the carding process;
- analyzing differences in terms of color in different sections and points of the tow; and
- providing a real-time process.

Future work will be addressed to the use of the colorimetric measurements for improving the carding process, e.g., by adding a feedback allowing a “quasi-real-time” change of the amount of pre-colored fibers to feed the carder. Other interesting improvements planned for the system are the use of spectral camera in the NIR spectrum to find possible correlations between invisible spectral reflectance and the presence of pollutants or undesired fibers (e.g., polyester or nylon in carded fabrics composed by wool 100%).

## Figures and Tables

**Table 1:** Composition and characteristics of the fabric used for reference.

Fabric Name (Company Code)	Number of Raw Materials	Original Recipe		CMC(2:1) Distance (Original Recipe)	Modified Recipe		CMC(2:1) Distance (Modified Recipe)
7460C	5	$\alpha_{1}$	0.09	0.5983	$\alpha_{1}$	0.130	0.3141
		$\alpha_{2}$	0.155		$\alpha_{2}$	0.120	
		$\alpha_{3}$	0.250		$\alpha_{3}$	0.240	
		$\alpha_{4}$	0.180		$\alpha_{4}$	0.205	
		$\alpha_{5}$	0.325		$\alpha_{5}$	0.305	

**Table 2:** Composition and characteristics of the fabric used for reference.

Sample #Number	CMC(2:1) Measurement #1	CMC(2:1) Measurement #5	CMC(2:1) Measurement #10	Mean Value for the 10 Readings
1	0.2933	0.3377	0.2324	0.2913
2	0.2857	0.2534	0.3267	0.2906
3	0.3199	0.2960	0.2402	0.2879
4	0.3306	0.3288	0.3287	0.3355
5	0.3383	0.3615	0.3445	0.3528
6	0.2569	0.3731	0.3422	0.3276
7	0.3255	0.3030	0.2122	0.2885
8	0.3214	0.2336	0.3187	0.2971
9	0.2376	0.2354	0.2988	0.2628
10	0.2302	0.2538	0.3458	0.2858
11	0.2947	0.3529	0.3716	0.2913
12	0.3732	0.2532	0.2481	0.2906
**Mean value**	**0.3006**	**0.2985**	**0.3008**	**0.3002**
**Standard Deviation**	**0.0428**	**0.0516**	**0.0534**	
